# Distinct Inflammation-Associated Microbiome Signatures in Pediatric Non-IgE-Mediated Food Allergy

**DOI:** 10.3390/ijms27167482

**Published:** 2026-08-21

**Authors:** Maria-Teodora Coșoreanu, Gratiela Gradisteanu Pircalabioru, Irina-Oana Lixandru-Petre, Mara-Ioana Ionescu, Andreea Ioan, Eliza-Elena Cinteză, Felicia Galoș

**Affiliations:** 1 Department of Pediatrics, Carol Davila University of Medicine and Pharmacy, 020021 Bucharest, Romania; maria-teodora.ilie@drd.umfcd.ro (M.-T.C.); andreea.berariu@drd.umfcd.ro (A.I.); eliza.cinteza@umfcd.ro (E.-E.C.); felicia.galos@umfcd.ro (F.G.); 2 Marie Curie Emergency Children’s Hospital, 041451 Bucharest, Romania; mara-ioana.ionescu@umfcd.ro; 3 Faculty of Biology, University of Bucharest, 030018 Bucharest, Romania; 4 eBio-Hub Centre of Excellence in Bioengineering, National University for Science and Technology, Politehnica Bucharest, 060042 Bucharest, Romania; irina.petre@upb.ro; 5 Department of Physiology-Neurosciences, Carol Davila University of Medicine and Pharmacy, 020021 Bucharest, Romania; 6 Department of Clinical Immunology, National Institute for Mother and Child Health Alessandrescu-Rusescu, 020395 Bucharest, Romania

**Keywords:** non-IgE-mediated food allergy, gut microbiome, 16S rRNA sequencing, fecal biomarkers, fecal calprotectin, eosinophil-derived neurotoxin, early-life exposures

## Abstract

Non-IgE-mediated food allergy is characterized by delayed gastrointestinal manifestations and the absence of reliable non-invasive biomarkers. Increasing evidence suggests that gut microbiota may contribute to this disease pathogenesis. The aim of this study was to characterize the gut microbiome composition in thirty pediatric patients diagnosed with non-IgE-mediated food allergy, in comparison to fifteen healthy controls children, and to investigate its association with fecal calprotectin, eosinophil-derived neurotoxin (EDN) and IgA. Gut microbiota profiling was performed by 16S rRNA gene sequencing targeting the V3–V4 region. Compared with healthy controls, higher mean relative abundances of *Bacteroides*, *Faecalibacterium*, *Alistipes*, *Parabacteroides*, and *Sutterella* were observed in patients. Conversely, healthy children showed higher mean relative abundances of *Pseudobutyrivibrio*, *Roseburia*, *Bifidobacterium*, *Collinsella*, *Clostridium*, *Eubacterium*, *Streptococcus*, and *Barnesiella*. Several genera (*Escherichia–Shigella*, *Agathobacter*, and *Enterococcus*/*Streptococcus*) were detected only in the allergy cohort. Shannon diversity was higher in patients compared to controls and in the subgroups of patients with elevated fecal calprotectin (*p* = 0.028), previous antibiotic exposure (*p* = 0.015), and atopic dermatitis (*p* = 0.021). Stratification according to inflammatory biomarkers identified a distinct inflammatory microbiome endotype characterized by increased fecal calprotectin and EDN together with enrichment of *Veillonellaceae* and depletion of *Bifidobacteriaceae* and *Lachnospiraceae*. Correlation analyses further revealed positive associations between *Veillonella* abundance and both fecal calprotectin and EDN. These findings suggest that pediatric non-IgE-mediated food allergy is characterized by distinct microbiome–inflammation relationships rather than a single dysbiotic signature.

## 1. Introduction

Non-IgE-mediated food allergy represents a group of immune-mediated adverse reactions to food antigens that develop independently of immunoglobulin E (IgE). Unlike classic IgE-mediated food allergy, which is characterized by rapid symptom onset, in non-IgE-mediated food allergy, the clinical manifestations are delayed, often developing 2 to 72 h after food ingestion, and predominantly involve the gastrointestinal tract [1,2]. The most common symptoms include diarrhea, hematochezia, vomiting, food refusal, failure to thrive, etc. [1,2].

Non-IgE-mediated food allergies encompass a spectrum of disorders, including food protein-induced enterocolitis syndrome (FPIES), food protein-induced allergic proctocolitis (FPIAP), food protein-induced enteropathy (FPE), eosinophilic esophagitis (EoE), food protein-induced gastroesophageal reflux disease (FPGORD), and food protein-induced constipation (FPC) [3]. These clinical entities are characterized by an overlap of symptoms, as a result of the broad spectrum of clinical manifestations of non-IgE-mediated food allergies [4]. In most cases, the onset occurs during infancy or early childhood, but they can also be diagnosed in later childhood or adolescence and are increasingly recognized in adults [5,6,7]. This group of conditions is characterized by significant diagnostic and management challenges due to their often subtle presentation, delayed symptom onset, reliance on clinical history and elimination diets for diagnosis, in the absence of specific, non-invasive diagnostic confirmatory tests [3]. Improved understanding of these disorders is essential in order to ensure early diagnosis and prevent unnecessary dietary restrictions.

The development of non-IgE-mediated food allergy is the result of a complex and incompletely elucidated interaction between the genetic background and various environmental factors acting during the intrauterine period, in the first months of life, and later on, leading to disruptive immune responses towards food proteins [4]. Increasing evidence suggests that interactions between dietary antigens, the intestinal immune system, and the gut microbiota play a critical role in disease development, but the precise mechanisms involved remain incompletely understood, leading to an increased need for additional research aiming to elucidate various etiopathogenic aspects with potential diagnostic and therapeutic applications [1,8,9].

The structural and functional maturation of the immune system is greatly influenced by the interactions with the intestinal microbiome, as the largest immunological tissue of the body is localized in the gastrointestinal tract, and the main source of immune microbial stimulation resides in the gut microbiota [10]. The microbial communities and their products are able to determine the differentiation and activation of various immune cells (regulatory T cells, natural killer T cells, B cells) and to influence the production of several cytokines and other immunological factors (IL-10, beta-defensin 3) [11,12,13]. Metabolic products of the microbiota are also involved, as butyrate and other fatty acids influence the maturation of the immune system by signaling macrophages, colonic T regulatory cells, and epithelial cells [14]. The complexity of the microbiome–immune interactions also resides in its bidirectional nature, as the composition of the microbiota is shaped by the immune system, one of the most important mechanisms being the selective inhibition of the growth of certain bacterial species by secretory IgA [11]. The concomitant and tightly linked development of the gut microbiome and immune system also ensures oral immune tolerance towards food antigens, through suppressive mechanisms of the immune responses [13]. The key role of the microbiome composition in influencing the fine regulation of the immune system during the first years of life is also reflected by the largely recognized contribution of early-life specific microbial exposures in reducing the risk of atopic, autoimmune, and inflammatory conditions [10]. On the other hand, environmental factors such as prenatal antibiotic exposure, maternal dietary habits, delivery by cesarian section, the absence of breastfeeding, and antibiotic exposure during the first months of life can negatively impact the composition of the gut microbiota, leading to an increased risk of allergic diseases [11,13]. It was shown that a delayed maturation of the gut microbiome in the first year of life was significantly associated with the presence of several allergic conditions at the age of five [15].

The significant inflammation localized in the digestive tract of non-IgE-mediated patients is also reflected in elevated levels of calprotectin and other fecal markers [16,17,18]. The involvement of gut microbiome alterations in the pathogenesis of non-IgE-mediated food allergies has been explored in several studies so far [9,19]. An increase in Pseudomonadota (formerly known as Proteobacteria) in the gut microbiota of patients diagnosed with non-IgE-mediated food allergy, and in the esophageal microbiota of EoE patients, was reported [9]. On the other hand, a decreased abundance of Bifidobacterium was identified in patients with non-IgE-mediated food allergy [20,21]. Results regarding lower alpha diversity of the gut microbiota were conflicting across various studies [22,23,24,25].

Given the limited understanding of non-IgE-mediated food allergy, the aim of this study was to explore various parameters in affected children. The main objectives were to describe the gut microbiome architecture in this cohort and to assess possible correlations between the microbiome composition and the levels of certain fecal markers reflecting intestinal inflammatory alterations (fecal calprotectin, eosinophil-derived neurotoxin (EDN), and fecal IgA). In order to further characterize the group of patients, perinatal, dietary, familial, environmental, and clinical particularities were described, and laboratory findings regarding nutritional status, intestinal inflammation, and permeability were reported.

## 2. Results

Thirty pediatric patients diagnosed with non-IgE-mediated food allergy were included in the analysis. The median age of the cohort was 2.5 years (interquartile range [IQR]: 1.0–6.75 years), reflecting a predominantly early childhood population. The sex distribution was balanced, with 16 females (53.3%) and 14 males (46.7%).

### 2.1. Early-Life, Familial, and Environmental Characteristics of the Cohort

Early-life exposures and familial characteristics were recorded for the pediatric patients and controls included in this study (Table 1). A positive family history of atopy was more frequently reported in patients compared to controls (50.0% vs. 13.3%, *p* = 0.023). While most patients were living in urban areas, all of the controls came from an urban background (73.3% vs. 100%, *p* = 0.038). Cesarean delivery occurred in 63.3% of the patients and in 66.7% of the controls (*p* = 1.000).

Antibiotic exposure during pregnancy was more frequently reported in patients compared to controls, but the level of statistical significance was not reached (30.0% vs. 13.3%, *p* = 0.288). Antibiotic administration in maternity occurred in 16.7% of children with non-IgE-mediated food allergy and in 0% of the controls. Smoking exposure during pregnancy was more frequent in controls compared to the patients, but the difference was not statistically significant (26.7% vs. 13.3%, *p* = 0.410).

Most children had been breastfed (86.7% of the patients and 80.0% of the controls, *p* = 0.670), but exclusive breastfeeding was reported in 36.7% of cases and in 33.3% of the healthy children (*p* = 1.000). The median duration of breastfeeding in patients was 4.5 months, while this information was not available for controls. The duration of breastfeeding was two months in four patients, while five patients were breastfed for three months, two patients for four months, one for five months, and one for six months. On the other hand, six patients were breastfed for a period between one and two years, and five other patients for more than two years, the maximum duration being 30 months. Most children also received milk formula in maternity (70.0% of patients and 66.7% of controls, *p* = 1.000).

Regarding family structure and household environment, 50.0% of children with non-IgE-mediated food allergy and 60.0% of controls had siblings (*p* = 0.752). A higher proportion of controls lived in households with pets, compared to the patients, but the level of statistical significance was not reached (46.7% vs. 26.7%, *p* = 0.200). A significantly higher proportion of fathers had completed higher education studies in controls compared to the patients (100% vs. 72.4%, *p* = 0.037). A higher education level was also more frequently completed by mothers from the control group compared to the patient group, but the difference was not significant (100% vs. 85.7%, *p* = 0.280). Given the limited sizes of the samples, the comparisons between controls and patients should be interpreted as exploratory. Overall, our patient group exhibited a substantial familial atopic predisposition and a moderate burden of early-life microbial exposures, consistent with risk profiles described in non-IgE-mediated food allergy. The high frequency of antibiotic exposure, combined with substantial familial atopy, may contribute to altered microbiota–immune interactions, favoring an allergic phenotype.

### 2.2. Clinical Manifestations

The presence of several clinical manifestations usually encountered in non-IgE-mediated food allergy was investigated in the group of patients in order to obtain a more complete characterization. (Table 2). These parameters were not systematically assessed in the healthy control group, as controls were included specifically for comparative microbiome analysis and were carefully selected to not present any allergic, gastrointestinal, or chronic inflammatory disease.

The most common symptom of non-IgE-mediated food allergy in our cohort was chronic diarrhea, reported in 40.0% of cases, while 20% of the patients presented multiple episodes of haematochezia. Vomiting and regurgitation were reported in 26.6% and 30.0% of the patients, respectively. Up to 36.6% of the children were diagnosed with failure to thrive, while 23.3% presented a history of food refusal. Constipation was the least frequent clinical manifestation, being reported in 6.6% of the children.

Extraintestinal manifestations were also present, with 40.0% of the patients associating atopic dermatitis.

Assessment of laboratory parameters revealed that 13.7% of the patients presented vitamin D deficiency (<20 ng/mL), while vitamin D insufficiency (21–29 ng/mL) was identified in up to 31.0% of the cases. Iron-deficiency anemia was detected in 10% of patients, consistent with the potential inflammatory profile and mucosal dysfunction associated with food allergy.

### 2.3. Fecal Markers Analysis

Fecal inflammatory markers reflected the burden of intestinal inflammation within the cohort (Table 3). Fecal inflammatory and intestinal-barrier biomarkers were assessed only in the patient group and were not systematically measured in healthy controls, who were included specifically as a reference cohort for microbiome analysis. Lactoferrin was elevated in 65.5% of patients, with the levels being increased in some of the cases in the context of breastfeeding. α-1 antitrypsin levels were elevated in 58.6% of children, suggesting mucosal inflammation associated with increased intestinal protein loss. Fecal calprotectin was elevated in 44.8% of patients, indicating neutrophil-driven intestinal inflammation. Although the mean fecal calprotectin level was 309.05 µg/g, the median value was considerably lower (76.30 µg/g), reflecting a right-skewed distribution due to high outliers in a subset of patients.

Fecal eosinophil-derived neurotoxin (EDN) was elevated in 33.3% of patients, with a mean value of 1200.82 ng/g and a median of 766.86 ng/g, supporting the presence of eosinophil-mediated intestinal inflammation in approximately one-third of cases.

Markers of mucosal immune activation were also altered. Fecal IgA levels were elevated in 54.5% of patients, with a mean concentration of 2318.83 µg/g and a median of 1990 µg/g, indicating heightened local immune responses.

In contrast, markers of intestinal barrier dysfunction were less prominently affected. Zonulin was elevated in only 10% of patients, with a mean value of 78.91 ng/mL and a median of 57.88 ng/mL, suggesting that increased intestinal permeability was not a universal feature of this cohort.

Overall, the biomarker profile of this cohort was characterized by predominant mucosal inflammatory activation, involving both neutrophilic and eosinophilic components, accompanied by evidence of intestinal protein loss. These findings suggest a model of chronic low-grade intestinal inflammation and mucosal immune dysregulation in pediatric non-IgE-mediated food allergy.

### 2.4. Gut Microbiome Analysis

Fecal microbiome profiling was performed using 16S rRNA gene sequencing on the Illumina MiSeq platform. After quality filtering and chimera removal, high-quality sequences were retained for downstream taxonomic analysis. Amplicon sequence variants were assigned to bacterial taxa using the SILVA reference database.

To investigate whether pediatric non-IgE-mediated food allergy could be associated with alterations in gut microbial composition, we compared the fecal microbiota of affected children with that of a healthy reference cohort. Comparative analyses included assessment of alpha diversity, taxonomic overlap, and differential relative abundance at the genus level.

**Figure 1:**
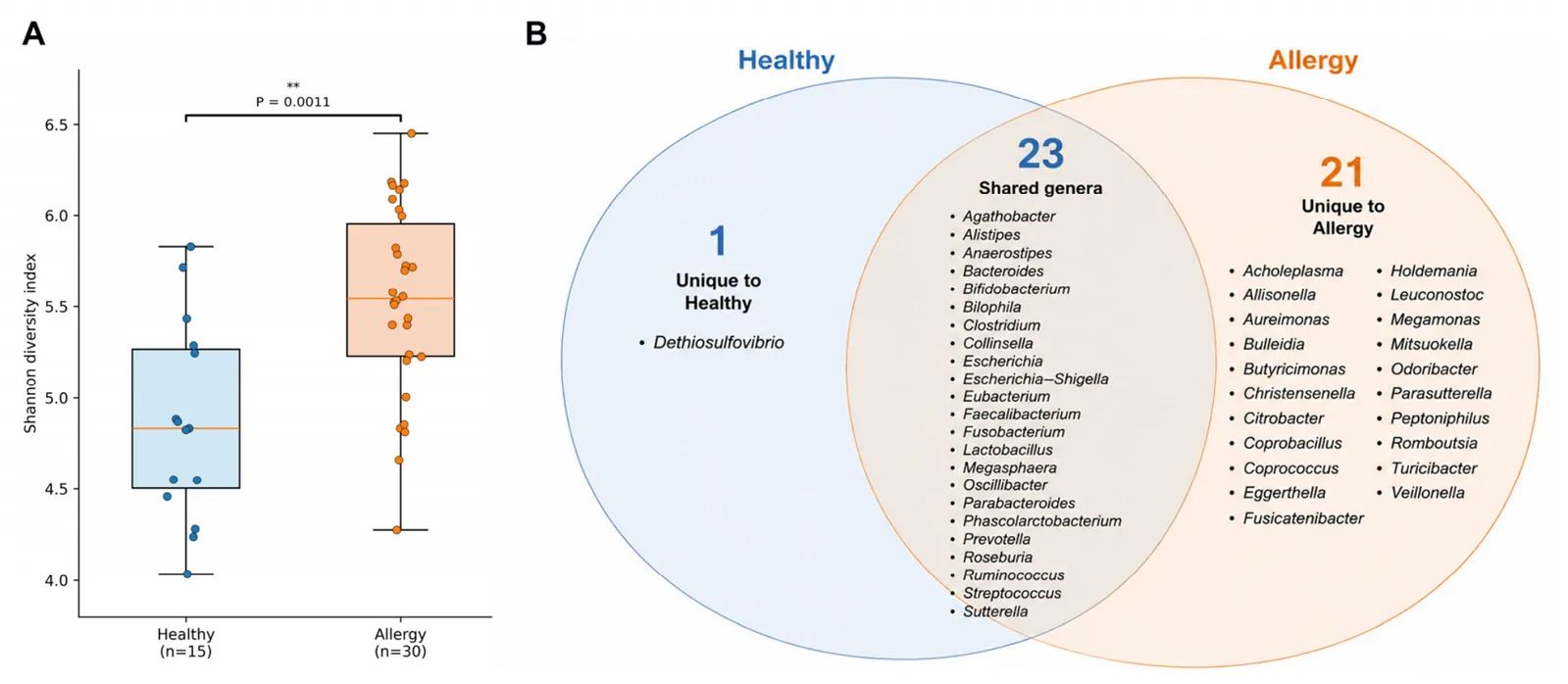
Comparison of gut microbiota diversity and taxonomic overlap between healthy controls and children with non-IgE-mediated food allergy. (**A**) Shannon diversity index in healthy controls (*n* = 15) and children with non-IgE-mediated food allergy (*n* = 30). Each point represents one participant. Differences between groups were evaluated using the two-sided Mann–Whitney U test. (**B**) Venn diagram showing the number of shared and group-specific bacterial genera. Only well-classified genera with a mean relative abundance ≥0.1% in at least one study group were included. ** *p* < 0.01.

Shannon diversity index was significantly higher among patients with non-IgE-mediated food allergy compared to controls (Figure 1A). Venn analysis of genus-level profiles demonstrated a substantial overlap between healthy controls and children with food allergy, indicating the presence of a conserved core gut microbiota (Figure 1B). Twenty-three genera were detected in both groups, including dominant commensals such as *Bacteroides*, *Faecalibacterium*, *Bifidobacterium*, *Roseburia*, *Ruminococcus*, *Parabacteroides*, *Alistipes*, *Prevotella*, and *Sutterella*. In contrast, 21 genera were identified exclusively in the allergy group, including *Veillonella*, *Parasutterella*, *Odoribacter*, *Megamonas*, *Christensenella*, *Eggerthella*, *Coprococcus*, *Fusicatenibacter*, and *Turicibacter*, suggesting increased microbial heterogeneity associated with allergic disease. Only one filtered genus, *Dethiosulfovibrio*, was detected exclusively in healthy controls. Overall, these findings indicate that food allergy is characterized primarily by the acquisition of additional low-abundance genera rather than the loss of the core gut microbiota.

Comparative analysis of genus-level relative abundance revealed differences in the microbial composition between healthy controls and children with non-IgE-mediated food allergy (Table 4). The allergy group showed higher mean relative abundances of *Bacteroides* (30.24% vs. 11.19%), *Faecalibacterium* (20.15% vs. 5.83%), *Alistipes*, *Parabacteroides*, *Escherichia*, and *Sutterella*. Conversely, healthy controls exhibited higher relative abundances of *Pseudobutyrivibrio*, *Roseburia*, *Bifidobacterium*, *Collinsella*, *Clostridium*, *Eubacterium*, *Streptococcus*, and *Barnesiella.* Several genera, including *Escherichia–Shigella*, *Agathobacter*, and *Enterococcus/Streptococcus*, were detected only in the allergy cohort, whereas others were absent or present at negligible abundance in healthy controls.

Despite these compositional differences, none of the investigated genera remained statistically significant after correction for multiple comparisons using the Benjamini–Hochberg FDR procedure (*q* > 0.05).

**Figure 2:**
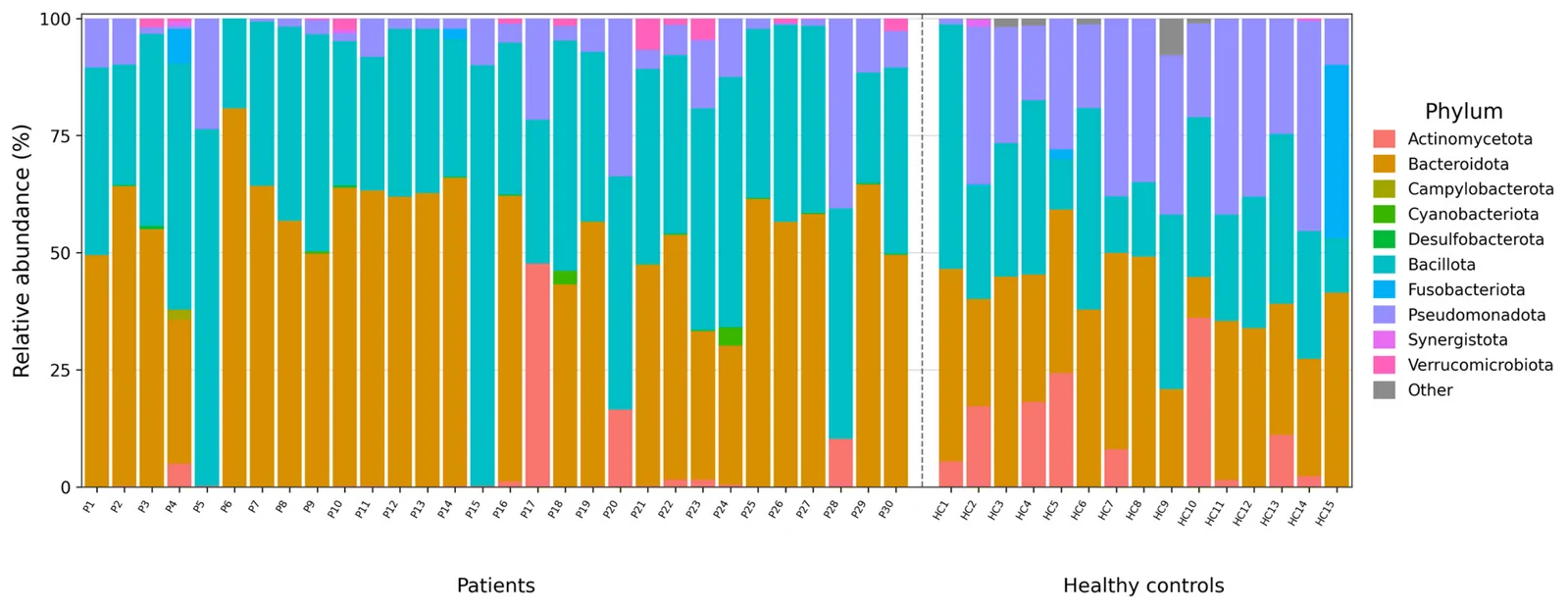
Phylum-level composition of the gut microbiota in children with non-IgE-mediated food allergy and healthy controls. Stacked bar plots show the relative abundance of bacterial phyla in individual patients with non-IgE-mediated food allergy (P1–P30) and healthy controls (HC1–HC15). Each bar represents one participant, and relative abundances were normalized to 100% per sample. The dashed vertical line separates the two study groups.

The phylum-level analysis showed that both healthy children and those with non-IgE-mediated food allergy were dominated by Bacteroidota and Bacillota, consistent with previous studies describing the core pediatric gut microbiota. No major phylum-level dysbiosis was evident, and considerable inter-individual variability was observed across both groups. These findings suggest that the microbial alterations associated with non-IgE-mediated food allergy are likely to involve specific bacterial genera or species rather than broad taxonomic shifts at the phylum level (Figure 2).

**Figure 3:**
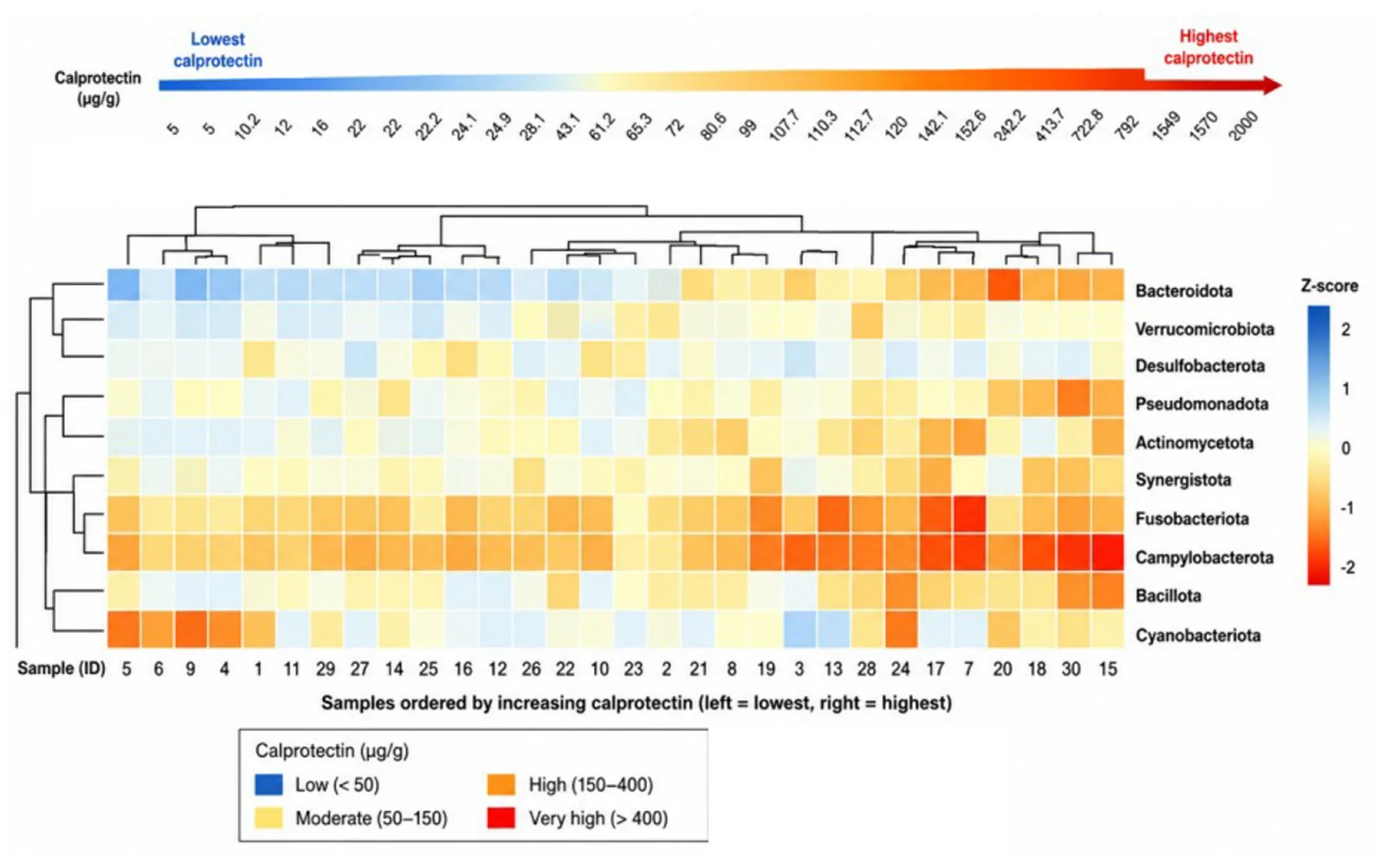
Phylum-level heatmap of the fecal microbiota ordered according to increasing fecal calprotectin concentrations in children with non-IgE-mediated food allergy. Heatmap showing the relative abundance of dominant bacterial phyla identified by 16S rRNA gene sequencing. Samples (columns) were arranged from left to right according to increasing fecal calprotectin concentrations, ranging from 5 to 2000 μg/g, allowing visualization of microbiome composition across an inflammatory gradient. Relative abundances were normalized using Z-scores, with blue indicating higher abundance and red indicating lower abundance relative to the cohort mean.

To investigate potential associations between intestinal inflammation and microbiome composition, samples were ordered according to increasing fecal calprotectin concentrations. The heatmap revealed substantial inter-individual variability in phylum-level microbiota composition across the cohort (Figure 3). Despite the presence of an inflammatory gradient, no single phylum demonstrated a consistent increase or decrease with rising fecal calprotectin concentrations.

Bacteroidota represented one of the most abundant phyla throughout the cohort and remained prevalent across both low- and high-calprotectin samples, suggesting preservation of this major anaerobic community regardless of inflammatory status. Similarly, Bacillota remained detectable in nearly all samples, although considerable variability in relative abundance was observed between individuals.

In contrast, Pseudomonadota exhibited localized enrichment in several samples characterized by higher fecal calprotectin concentrations, particularly among individuals with moderate-to-severe inflammatory activity. However, this pattern was not universal, indicating that elevated intestinal inflammation was not uniformly associated with expansion of this phylum. Actinomycetota, Desulfobacterota, and Verrucomicrobiota also displayed sporadic enrichments in individual samples but did not exhibit a clear association with fecal calprotectin levels.

To further explore whether the observed microbial heterogeneity was associated with clinical and inflammatory characteristics, alpha diversity analyses were performed using the Shannon diversity index and compared across patient subgroups defined by biomarker status and clinical variables.

Alpha diversity metrics were calculated to assess microbial richness and community structure within the cohort. Given the limited sample size beta diversity group comparisons using PERMANOVA were not performed. Microbiome analyses were primarily descriptive and hypothesis-generating.

**Figure 4:**
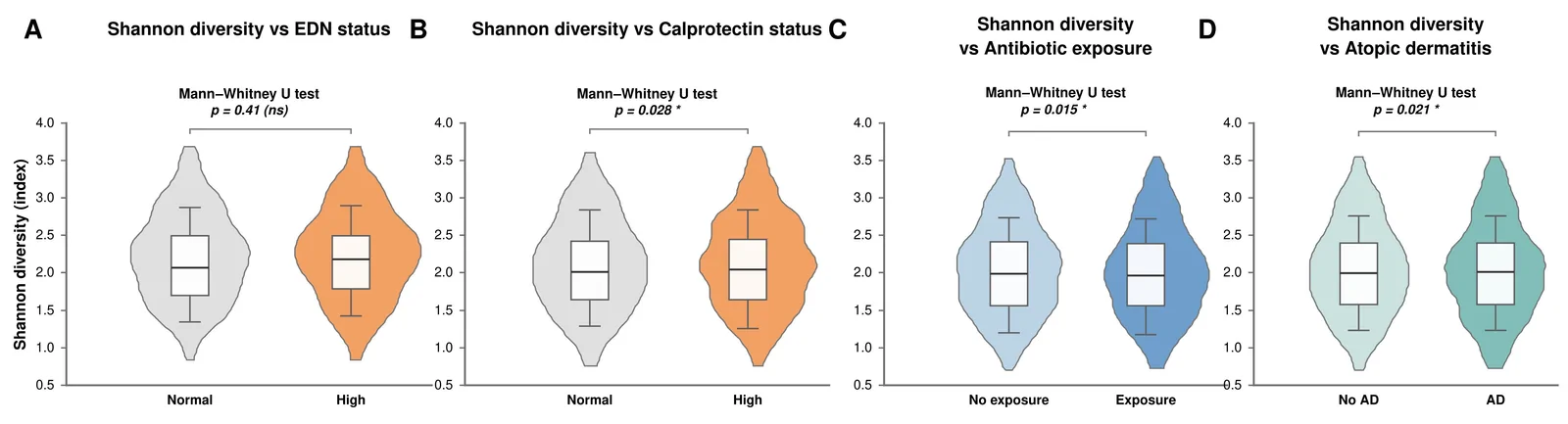
(**A**) Comparison of Shannon diversity between children with normal and elevated fecal eosinophil-derived neurotoxin (EDN) concentrations (Mann–Whitney U test, *p* = 0.41). (**B**) Comparison of Shannon diversity according to fecal calprotectin status (Mann–Whitney U test, *p* = 0.028). (**C**) Comparison of Shannon diversity between children with and without antibiotic exposure (Mann–Whitney U test, *p* = 0.015). (**D**) Comparison of Shannon diversity according to atopic dermatitis (AD) status (Mann–Whitney U test, *p* = 0.021). Violin plots illustrate the distribution of Shannon diversity values within each group, while embedded boxplots indicate the median and interquartile range (IQR). Statistical significance was assessed using the Mann–Whitney U test, with *p* < 0.05 considered significant. * *p* < 0.05.

Alpha diversity analyses demonstrated that overall gut microbial diversity was associated with several clinical and inflammatory parameters (Figure 4A–D). While no significant differences in Shannon diversity were observed according to EDN status (*p* = 0.41), children presenting elevated fecal calprotectin concentrations exhibited significantly higher microbial diversity than those with normal calprotectin levels (*p* = 0.028). Similarly, antibiotic exposure was associated with increased Shannon diversity (*p* = 0.015), and patients with atopic dermatitis displayed significantly higher diversity compared to children without dermatological manifestations (*p* = 0.021).

**Figure 5:**
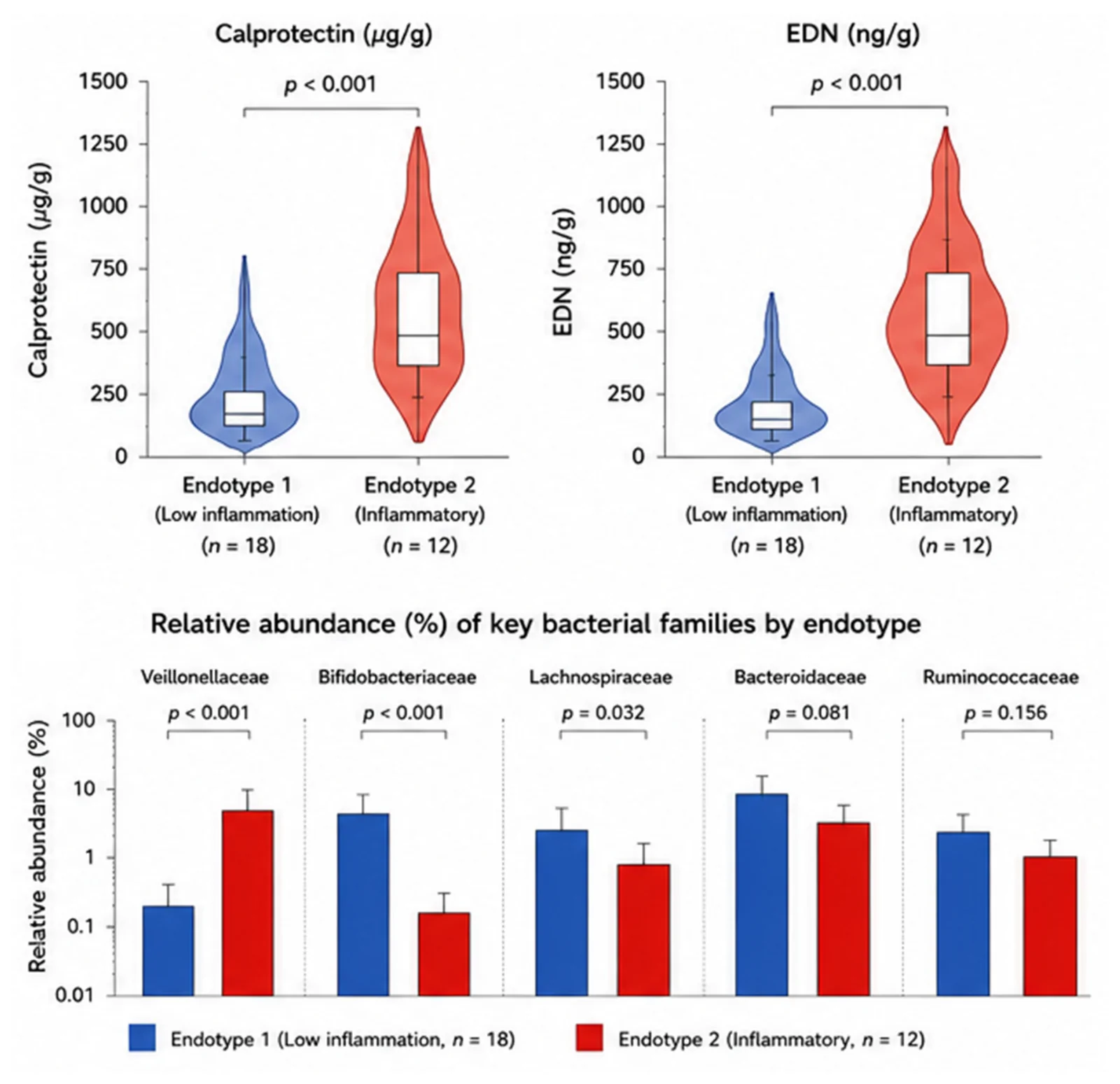
Identification of inflammation-associated microbiome endotypes in pediatric non-IgE-mediated food allergy. Children were stratified into two microbiome-associated inflammatory endotypes based on fecal inflammatory biomarker profiles: Endotype 1 (low inflammation, *n* = 18) and Endotype 2 (inflammatory, *n* = 12). Upper panels show violin plots with embedded boxplots comparing fecal calprotectin and EDN concentrations between endotypes. Patients assigned to the inflammatory endotype exhibited significantly higher levels of both calprotectin and EDN compared with the low-inflammation endotype (Mann–Whitney U test, *p* < 0.001 for both comparisons). Lower panels illustrate the relative abundance of key bacterial families associated with each endotype. The inflammatory endotype was characterized by a significant enrichment of *Veillonellaceae* (*p* < 0.001) and a significant depletion of *Bifidobacteriaceae* (*p* < 0.001) and *Lachnospiraceae* (*p* = 0.032).

In order to further investigate whether intestinal inflammation was associated with specific microbial patterns, patients were stratified according to fecal inflammatory biomarker levels, and the relative abundance of dominant bacterial families was compared (Figure 5).

Children presenting elevated inflammatory biomarkers (endotype 1-inflammatory) exhibited significantly higher concentrations of both fecal calprotectin and eosinophil-derived neurotoxin (EDN), indicating increased intestinal inflammatory activity and eosinophilic involvement (*p* < 0.001 for both comparisons). These inflammatory changes were accompanied by distinct alterations in gut microbiota composition. Among the bacterial families analyzed, *Veillonellaceae* showed the strongest positive association with inflammation, displaying significantly higher relative abundance in patients with elevated inflammatory biomarkers (*p* < 0.001). In contrast, *Bifidobacteriaceae* abundance was significantly reduced in the inflammatory group (*p* < 0.001). A similar trend was observed for *Lachnospiraceae*, which were also significantly depleted in children with higher inflammatory burden (*p* = 0.032).

Although *Bacteroidaceae* and *Ruminococcaceae* tended to be less abundant in patients with elevated inflammatory markers, these differences did not reach statistical significance (*p* = 0.081 and *p* = 0.156, respectively).

Collectively, these findings indicate that intestinal inflammation in pediatric non-IgE-mediated food allergy is associated with specific compositional shifts within the gut microbiota, characterized by enrichment of *Veillonellaceae* and depletion of bacterial families generally linked to intestinal homeostasis.

**Figure 6:**
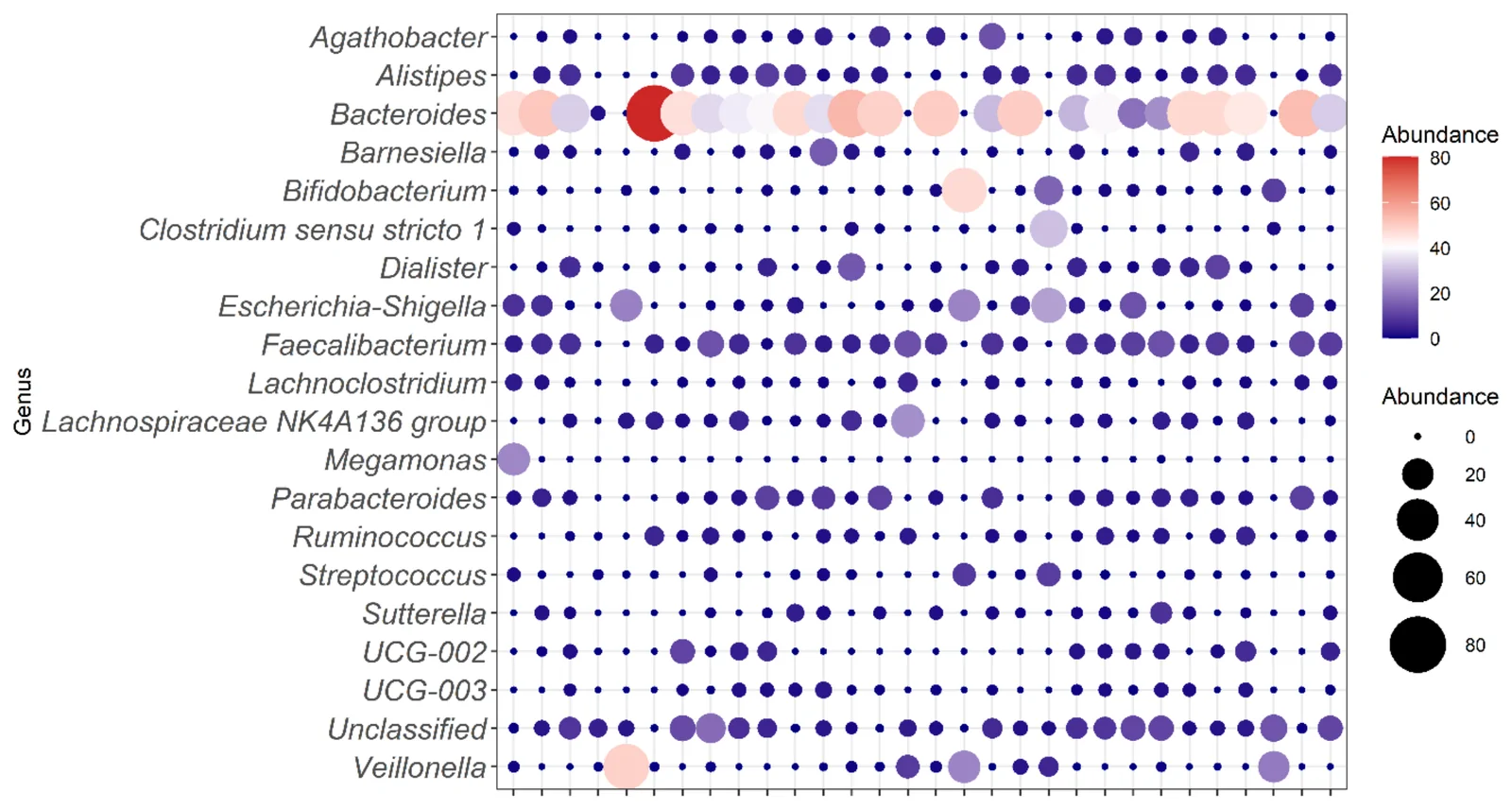
Genus-level microbial composition of fecal samples from children with non-IgE-mediated food allergy. Bubble plot illustrating the relative abundance of the most prevalent bacterial genera identified by 16S rRNA gene sequencing. Each column represents an individual fecal sample and each row corresponds to a bacterial genus. Bubble size and color intensity are proportional to relative abundance, with larger and darker red circles indicating higher abundance.

To obtain a more detailed characterization of the gut microbial communities, taxonomic profiles were further analyzed at the genus level (Figure 6). Considerable inter-individual variability was observed across the cohort, with several genera exhibiting marked differences in relative abundance between samples.

The gut microbiota was predominantly characterized by members of the genera *Bacteroides*, *Faecalibacterium*, *Alistipes*, *Parabacteroides*, and *Agathobacter*, which were detected in the majority of samples. Among these taxa, *Bacteroides* represented the most abundant genus, reaching relative abundances exceeding 80% in certain individuals. However, substantial variation in *Bacteroides* abundance was observed across the cohort, indicating considerable heterogeneity in microbial community structure among patients with non-IgE-mediated food allergy.

Several genera commonly associated with short-chain fatty acid production and intestinal homeostasis, including *Faecalibacterium*, *Agathobacter*, *Ruminococcus*, and members of the *Lachnospiraceae NK4A136* group, were identified across multiple samples, although their abundance varied considerably between individuals. In contrast, potentially pro-inflammatory taxa such as *Escherichia-Shigella*, *Veillonella*, *Dialister*, and *Streptococcus* were enriched in a subset of patients but were absent or present at low abundance in others.

Notably, *Veillonella* demonstrated pronounced enrichment in several samples, whereas it was nearly absent in others, suggesting a potential contribution to disease heterogeneity. Likewise, increased abundance of *Escherichia-Shigella* was detected in specific individuals, consistent with previous reports linking Enterobacterales expansion to intestinal inflammation and microbial dysbiosis.

**Figure 7:**
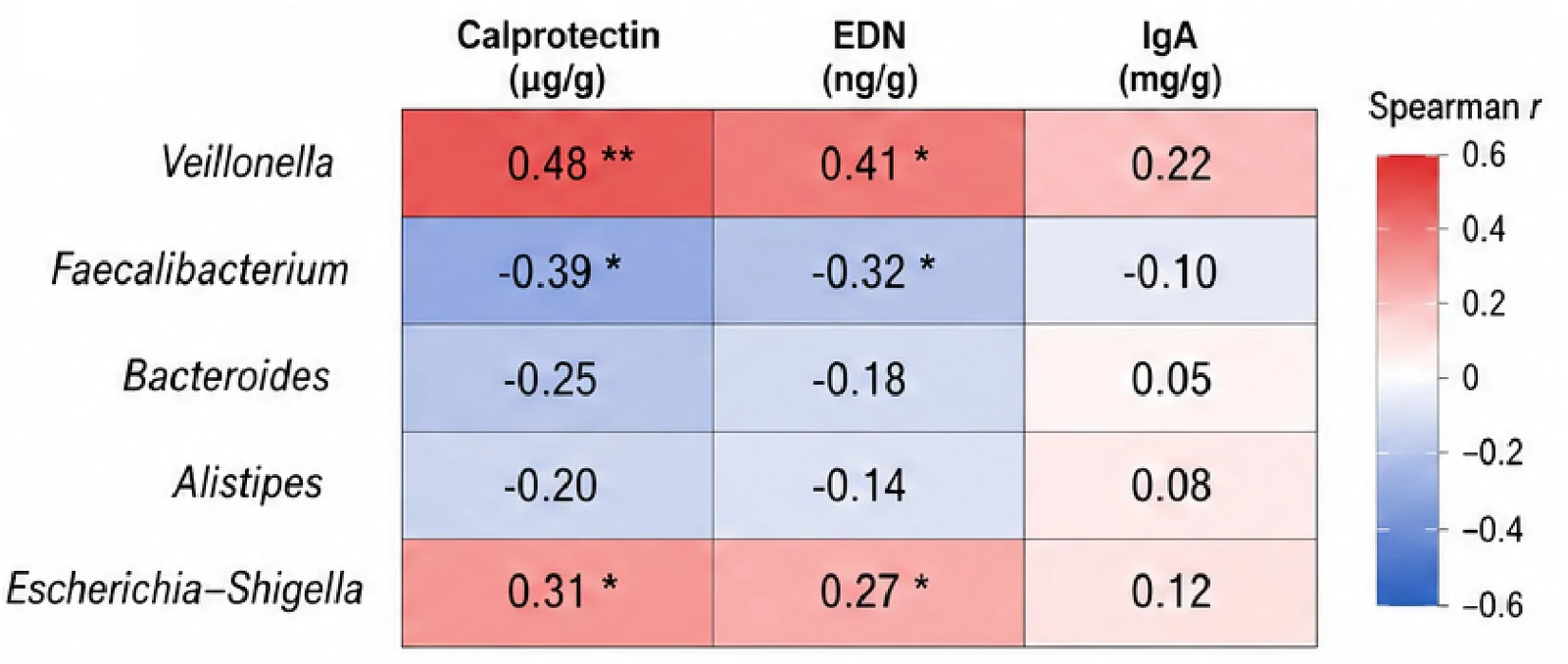
Correlations between dominant bacterial genera and fecal inflammatory biomarkers in pediatric non-IgE-mediated food allergy. Heatmap showing Spearman correlation coefficients (ρ) between the relative abundance of selected bacterial genera and fecal inflammatory biomarkers, including fecal calprotectin, eosinophil-derived neurotoxin (EDN), and immunoglobulin A (IgA). Positive correlations are represented in red and negative correlations in blue, with color intensity proportional to the correlation coefficient. Significant associations are indicated by asterisks (* *p* < 0.05, ** *p* < 0.01).

Spearman correlation analysis revealed significant associations between several dominant bacterial genera and fecal inflammatory biomarkers in children with non-IgE-mediated food allergy (Figure 7). Among all taxa examined, *Veillonella* demonstrated the strongest positive association with intestinal inflammation. The relative abundance of *Veillonella* correlated positively with both fecal calprotectin (*ρ* = 0.48, *p* < 0.01) and EDN (*ρ* = 0.41, *p* < 0.05), indicating that increased abundance of this genus was associated with greater neutrophilic and eosinophilic intestinal inflammatory activity. In contrast, *Faecalibacterium* showed significant negative correlations with fecal calprotectin (*ρ* = –0.39, *p* < 0.05) and EDN (*ρ* = –0.32, *p* < 0.05), suggesting a potential protective role against intestinal inflammation. *Escherichia–Shigella* was also positively associated with inflammatory biomarkers, displaying significant positive correlations with fecal calprotectin (*ρ* = 0.31, *p* < 0.05) and EDN (*ρ* = 0.27, *p* < 0.05). Conversely, *Bacteroides* and *Alistipes* exhibited weak negative correlations with inflammatory markers, although these associations did not reach statistical significance. No significant correlations were observed between the analyzed bacterial genera and fecal IgA concentrations, suggesting that microbial associations were more closely linked to inflammatory activity than to local mucosal immunoglobulin production.

## 3. Discussion

### 3.1. Intestinal Microbiome Alterations in Patients with Non-IgE-Mediated Food Allergy

Alpha diversity, taxonomic composition, and genus-level differential relative abundance were compared between patients with non-IgE-mediated food allergy and a healthy reference cohort. While the majority of dominant genera were shared between groups, several taxa were detected exclusively in the allergy cohort, suggesting that the disease is associated with the acquisition of specific microbial signatures rather than a complete disruption of the core gut microbiota.

In the group of patients, higher mean relative abundances were identified for several species, such as *Bacteroides* (30.24% vs. 11.19%) and *Faecalibacterium* (20.15% vs. 5.83%). On the other hand, in the control group, higher relative abundances of *Pseudobutyrivibrio*, *Roseburia*, *Bifidobacterium*, *Collinsella*, *Clostridium*, *Eubacterium*, *Streptococcus*, and *Barnesiella* were detected. Although compositional differences were detected, statistical significance was not maintained for any of the investigated genera after applying the Benjamini–Hochberg FDR correction for multiple testing (*q* > 0.05). This outcome is likely attributable to the small size of the healthy control cohort and the high degree of inter-individual variation in the pediatric gut microbiome. Therefore, these observations should be considered exploratory and interpreted with caution.

Although alpha diversity remained relatively preserved across the cohort, significant differences were observed according to fecal calprotectin status, antibiotic exposure, and atopic dermatitis, indicating that clinical factors may contribute to shaping microbial community structure These findings also align with the observations from the comparison with the healthy control group, indicating that food allergy is characterized primarily by the acquisition of additional low-abundance genera rather than the loss of the core gut microbiota.

A particularly noteworthy finding was the strong association between *Veillonellaceae* abundance and inflammatory biomarkers. *Veillonellaceae* demonstrated significant positive correlations with EDN and fecal calprotectin, suggesting a potential link between this bacterial family and intestinal inflammation. Members of *Veillonellaceae* are lactate-utilizing anaerobes that have previously been described to be implicated in inflammatory bowel disease, asthma, eosinophilic gastrointestinal disorders, and food allergy, as their ability to metabolize lactate and generate propionate may influence local immune responses and alter microbial cross-feeding networks, potentially contributing to mucosal immune activation [26,27,28,29,30].

Conversely, *Bifidobacteriaceae* displayed consistent negative associations with inflammatory biomarkers. This observation is consistent with previous studies showing that *Bifidobacterium* species promote epithelial barrier integrity, stimulate regulatory immune responses, and suppress inflammatory signaling pathways, reduced abundance of these beneficial microorganisms being repeatedly reported in allergic and inflammatory disorders, supporting their potential protective role in maintaining intestinal homeostasis [20,21,30,31].

The observed variability in taxonomic composition highlights the heterogeneous nature of gut microbial communities in pediatric non-IgE-mediated food allergy. It is possible that differences in host characteristics, environmental exposures, dietary patterns, or disease phenotype contribute to the variability observed across patients. Even when major taxonomic groups remain detectable, subtle functional alterations in microbial metabolism or host–microbe signaling may influence intestinal immune responses, although such mechanisms cannot be inferred directly from 16S rRNA gene sequencing data.

Several studies explored the characteristics of the gut microbiome in patients diagnosed with food allergy, in the context of a possible role of pathological microbiota in the development of these disorders [9,32]. Alterations in the composition of the intestinal microbiome have been reported, with various specific findings, while the causality remains uncertain [9,22]. Antibiotic exposure, prematurity, and cesarean birth are associated with intestinal microbiome alterations consisting in a reduction of Bacteroidota [23,33,34].

Lower alpha diversity has been identified in several studies, whereas others did not find a change in alpha diversity in children with food allergy [22,23,24,25]. A decreased abundance of *Bifidobacterium* was reported in several studies including children with non-IgE-mediated CMPA [20,21]. A reduced abundance of *Bifidobacterium*, *Collinsella*, *and Limosilactobacillus* and an increased abundance *of Hungatella*, *Veillonella*, *Citrobacter*, and *Ruminococcus gnavus* were reported in a Turkish study including children with non-IgE-mediated CMPA [30]. In a group of patients diagnosed with FPIES, Pseudomonadota (especially *Escherichia-Shigella*) and *Porphyromonadaceae* were significantly more frequent than in controls [35]. Pseudomonadota enrichment reflects an imbalanced gut microbiome structure and can trigger inflammatory responses, being influenced by environmental factors and diet [9]. Another study assessing the microbiota profile of children with FPIES found a higher abundance of *Lachnospiraceae* and a lower proportion of *Lactobacillaceae* and *Ruminococcaceae* [36].

The clinical relevance of assessing microbiome changes in patients with non-IgE-mediated conditions is supported by the emerging evidence of the therapeutic utility of adding probiotic strains (*Lactobacillus GG*, *Bifidobacterium breve M-16V*) to the composition of extensively-hydrolysed and amino-acid-based formulas used in the management of these children [37,38,39,40].

### 3.2. Fecal Markers and Non-IgE-Mediated Food Allergy

The presence of intestinal inflammation and increased permeability associated with food allergy in our group of patients was reflected by the increased levels of the assessed fecal markers. More than half of the patients in our study had elevated levels of fecal calprotectin, alpha-1-antitrypsin, lactoferrin, and fecal IgA, while EDN was elevated in one-third of patients, and fecal zonulin in just 10%. In pediatric populations, the levels of fecal markers such as calprotectin, lactoferrin, and EDN can be influenced by several factors, including age, diet, breastfeeding status, microbial exposures, and intercurrent infections. Therefore, these markers should be interpreted with caution as supportive indicators of mucosal immune activity rather than as disease-specific diagnostic markers for non-IgE-mediated food allergy.

Given the fact that currently no specific diagnostic tests exist for non-IgE-mediated food allergy, multiple studies assessed the utility of certain markers in supporting the diagnosis of this allergic pathology, fecal markers being among the most studied ones and the most commonly tested in clinical practice. Calprotectin, representing a calcium- and zinc-binding protein found in neutrophils and monocytes, possessing antimicrobial and immunomodulatory properties, was the first fecal marker proposed to be associated with non-IgE-mediated food allergy [41,42]. A systematic review and meta-analysis concluded that fecal calprotectin levels were significantly higher in children with CMPA [43]. However, mainly due to the high variability in concentration in children and conflicting results across multiple studies, fecal calprotectin failed to become a reliable and validated diagnostic tool for non-IgE-mediated food allergy [44,45,46,47]. Fecal calprotectin also showed potential as a marker of disease evolution in children with CMPA, as significant decreases in values were reported after following a milk-elimination diet [17,41,42,48,49].

A series of studies reported increased levels of EDN (one of the proteins with antimicrobial effects, released from the granules of eosinophils) in children with non-IgE-mediated food allergy [45,50,51]. In children with FPIAP, when the diagnostic performance of fecal calprotectin, EDN, and TNF-α was assessed, the best result was achieved for the combination of EDN and fecal calprotectin [51]. Additionally, in a study by Wada et al., an increase in EDN levels was reported after ingestion of the culprit foods in patients with FPIES [52]. On the other hand, Roca et al. found no significant difference in EDN levels before and after a 1-month elimination diet [47].

Fecal zonulin, a modulator of intercellular tight junctions in the digestive tract, representing a marker of increased intestinal permeability, also showed a potential utility in the diagnosis of children with CMPA [42]. Moreover, patients with milk-induced FPIAP showed a significant decrease in this marker after a 4-week milk-elimination diet [42]. The utility in diagnosing food allergy was less investigated in the case of other fecal markers such as α-1 antitrypsin, β-defensin, tumor necrosis factor-α (TNF-α), fecal IgA, and eosinophilic cationic protein (ECP), which were also proposed as potential tools in the diagnosis of non-IgE-mediated food allergy [18].

### 3.3. Risk Factors Associated with Non-IgE-Mediated Food Allergy

A significantly higher percentage of the patients presented a positive history of allergic disease in parents or siblings compared to controls (50.0% vs. 13.3%, *p* = 0.023), consistent with the well-established role of genetic predisposition in the immune dysregulation underlying atopic conditions. A large number of studies reported the involvement of a positive family history of atopy in increasing the risk of food allergy, suggesting the key role of the genetic background in the development of the disease [53,54,55,56,57,58,59,60,61,62]. It is important to mention that we previously reported a link between genetic factors and the development of non-IgE-mediated food allergy in a larger cohort of Romanian children that also included the patients from the present study [63]. More specifically, a significant association was identified for the first time between *IL13* single-nucleotide gene polymorphism rs1800925 and the risk of non-IgE-mediated food allergy [63].

Among the environmental risk factors assessed in our patients, the urban background and the presence of a paternal higher level of education were significantly more frequently reported in controls. Although the frequency of maternal use of antibiotics during pregnancy was more than twice as high in patients, the level of statistical significance was not reached. No significant differences between the patient and control groups were observed for maternal age at birth, smoking exposure during pregnancy, premature birth, type of delivery, antibiotic treatment in maternity, formula feeding in maternity, breastfeeding, the presence of siblings, income, or pets at home. Given the limited sizes of the samples, these results should be cautiously interpreted.

Previous studies investigating environmental risk factors for non-IgE–mediated food allergy have reported heterogeneous results. An older age of the mother at the time of birth, prematurity, cesarian delivery, a reduced duration of breastfeeding, exclusive formula feeding, the absence of pets at home, smoking exposure, low socioeconomic status, antibiotic exposure during pregnancy or the first years of life and vitamin D deficiency were all reported to be associated with an increased risk of non-IgE-mediated food allergy, although contradictory results across different studies are present for some of these factors [1,53,54,55,64,65,66,67]. Antibiotic exposure during pregnancy was found to be associated with an increased risk of food allergy in children [56,57,68,69,70]. However, in a meta-analysis assessing the associations between maternal antibiotic exposure during pregnancy and the risk of several allergic diseases in childhood, no significant association was found [71]. Exposure to smoking during pregnancy and in the first year of life was also associated with food allergy [64,72], while in a Polish study, no correlation was found [55]. The link between cesarean delivery and an increased risk of food allergy was confirmed in some studies, whereas in others no association was found [55,65,73,74,75]. Research assessing the protective effect of breastfeeding against food allergy yielded contradictory results, some of the reasons probably being the variable composition of human milk, gene–environment interactions, and the small number of studies conducted so far [53,55,76,77].

The high degree of variability in the study results regarding environmental risk factors associated with non-IgE-mediated food allergy points towards complex gene–environmental interactions starting in the intrauterine period of development and leading to immune dysregulation. Therefore, further research is needed to explore the risk factors and etiopathogenic mechanisms of this type of allergic pathology.

### 3.4. Novelty

The novelty of the present study lies in the integrated characterization of gut microbiota composition, fecal inflammatory biomarkers, and clinical manifestations in children with non-IgE-mediated food allergy. While previous studies have primarily focused on describing taxonomic alterations associated with food allergy, our work extends these observations by linking microbial signatures to objective markers of intestinal inflammation, including fecal calprotectin and eosinophil-derived neurotoxin (EDN). By ordering microbiome profiles along an inflammatory gradient and performing correlation analyses between dominant bacterial taxa and inflammatory biomarkers, we identified inflammation-associated microbial signatures characterized by enrichment of *Veillonellaceae* and depletion of *Bifidobacteriaceae* and *Lachnospiraceae*. Furthermore, we demonstrate associations between microbial diversity and clinically relevant factors such as antibiotic exposure and atopic dermatitis, providing additional insight into disease heterogeneity. Collectively, these findings suggest that pediatric non-IgE-mediated food allergy is associated with distinct microbiome–inflammation relationships rather than a single dysbiotic pattern, highlighting potential microbial biomarkers that may contribute to future patient stratification and personalized therapeutic approaches.

### 3.5. Limitations

This study has several limitations that should be considered when interpreting the findings. Although the inclusion of a healthy reference cohort enabled exploratory comparisons with children with non-IgE-mediated food allergy, the relatively small number of healthy controls limited the statistical power to detect differences after correction for multiple testing. Moreover, the relatively small sample sizes increase the possibility of both type I and type II errors, thereby reducing the ability to detect modest associations and potentially inflating the uncertainty surrounding effect estimates. Consequently, the comparative analyses should be considered hypothesis-generating rather than definitive evidence of disease-associated microbial biomarkers. In addition, the cross-sectional design precludes causal inference regarding the relationship between microbiome alterations and intestinal inflammation, and longitudinal studies will be necessary to determine whether the identified microbial signatures precede disease onset or reflect disease progression.

The age range of the cohort was relatively wide, and it is well established that gut microbiome composition undergoes significant maturation during early childhood. Differences between infant and preschool microbiota may therefore contribute to part of the inter-individual variability observed in this study. Future investigations, including larger cohorts, will be necessary to assess age-related effects on microbiome composition in non-IgE-mediated food allergy. Furthermore, several environmental and therapeutic factors that influence early-life microbiota development, such as antibiotic exposure, type of delivery, or formula feeding, may have affected the observed associations between inflammatory status and microbial composition. Although our findings identify microbial taxa associated with inflammation, the observational nature of the study precludes definitive conclusions regarding whether these taxa represent inflammation-specific signatures or, in part, reflect differences in these underlying exposures.

Another limitation of the study was that the microbiome analyses were conducted at the family level, which may obscure strain-level variation and functional heterogeneity within taxa. Given that microbial metabolic capacity and immunomodulatory effects often vary at the strain level, taxonomic resolution at the family scale may underestimate biologically meaningful differences. It is important to note that V3–V4 16S sequencing is characterized by several limitations regarding functional inference and may influence the interpretation of microbiome structure. The results should be interpreted as exploratory and hypothesis-generating, in the context of the heterogeneity of the existing literature. Previous studies investigating the gut microbial alterations associated with food allergy have reported variable results, likely reflecting differences in study populations, inclusion criteria, disease severity, timing of biological sample collection, sequencing platforms, bioinformatic pipelines, and statistical approaches.

### 3.6. Future Directions

Future research should aim to address the above-mentioned limitations through the inclusion of larger and longitudinal cohorts, enabling more robust multivariable modeling and temporal assessment of microbiome–host interactions. Future prospective, longitudinal studies with comprehensive data collection and appropriate adjustment for key confounders are also needed to determine whether these microbial signatures represent independent features of inflammatory endotypes or surrogate markers of underlying exposures. Higher-resolution microbiome profiling, including strain-level characterization and shotgun metagenomic sequencing, metatranscriptomics, metabolomics, and targeted experimental validation, would provide deeper insight into functional potential. Functional pathway prediction analyses, particularly those focused on short-chain fatty acid biosynthesis and other immunoregulatory metabolic pathways, may further clarify mechanistic links between microbial activity and disease expression.

## 4. Materials and Methods

### 4.1. Study Group

A total of 30 pediatric patients diagnosed with non-IgE-mediated food allergy were included in this study. All children were recruited from Marie Curie Emergency Children’s Hospital, Bucharest, Romania.

Inclusion in the patient group was based on the coexistence of the following aspects:

1. The presence of a well-documented clinical history characterized by consistent delayed reactions—such as failure to thrive, vomiting, chronic diarrhea, hematochezia, and chronic abdominal pain—with symptoms reproducibility upon repeated exposures to the triggering food allergens.
2. The presence of a significant improvement or complete remission of the clinical manifestations at the 1-month follow-up after starting the food elimination diet.
3. The presence of informed consent given by the parents for inclusion in the study.

Exclusion criteria:

1. A high suspicion for the presence of alternative diagnoses or physiologic causes of the symptomatology, given all the information gathered based on the well-documented clinical history, present clinical assessment, and laboratory findings.
2. The impossibility of gathering all the relevant variables included in the study design.

In all of the patients, the consistency of the well-documented clinical history, combined with the unequivocal improvement following dietary elimination, was considered sufficient to establish the diagnosis. Due to logistical and ethical considerations and the lack of universally accepted protocols for oral food challenge (OFC) tests, performing them in the patients included in the study was not feasible.

In addition to the 30 children diagnosed with non-IgE-mediated food allergy, a healthy reference cohort comprising 15 age-matched children without allergic, gastrointestinal, or chronic inflammatory diseases was included for comparative microbiome analysis. The same sample collection procedures, DNA extraction protocol, and 16S rRNA sequencing workflow as for the patient cohort were used for the healthy controls. Owing to the limited number of controls, comparisons with the healthy cohort were considered exploratory and intended to provide biological context rather than establish diagnostic biomarkers.

The study was conducted in accordance with the Declaration of Helsinki and approved by the institutional Ethics Committee of Marie Curie Emergency Children’s Hospital and the University of Bucharest. Written informed consent was obtained from the parents or legal guardians of all participating children prior to enrollment.

### 4.2. Data Collection

Data regarding early-life exposures and familial characteristics were obtained using detailed questionnaires filled in by the parents. Laboratory parameters—including complete blood count, vitamin D levels, fecal calprotectin, lactoferrin, zonulin, eosinophil-derived neurotoxin (EDN), fecal IgA, and α1-antitrypsin—were determined in the laboratory unit of Marie Curie Emergency Children’s Hospital, and data were extracted from digital patient records. Hemoglobin levels were determined using spectrophotometry. For the measurement of vitamin D levels, electrochemiluminescence techniques were used. Enzyme Immunoassay (EIA) was performed for determining the levels of fecal lactoferrin, zonulin, eosinophil-derived neurotoxin (EDN), fecal IgA, and α1-antitrypsin, while the method used for fecal calprotectin was chemiluminescence.

### 4.3. Statistical Analysis

Categorical variables are reported as absolute and relative frequencies. For perinatal and environmental data, normally distributed continuous variables are reported as mean ± standard deviation (SD), while non-normally distributed variables are presented as median with interquartile range (IQR). In order to ensure a more detailed characterization of the fecal markers, able to better reflect the levels detected in our cohort, both mean ± standard error of the mean (SEM) and median with IQR were reported.

Demographic, perinatal, and familial characteristics were compared between patients and controls. Categorical variables were analyzed using Fisher’s exact test because of the small sample size and low expected cell frequencies, while maternal age was compared using Welch’s independent-samples *t*-test. Percentages were calculated using the available observations for each variable.

The distribution of continuous variables, including Shannon diversity values, was assessed using the Kolmogorov–Smirnov normality test. Because Shannon diversity values did not satisfy the assumption of normality and the analyzed subgroups were relatively small, comparisons between independent groups were performed using the two-sided Mann–Whitney U test. This test was used to compare Shannon diversity between children with normal and elevated fecal EDN or calprotectin concentrations, children with and without previous antibiotic exposure, and children with and without atopic dermatitis. Previous antibiotic exposure was defined as antibiotic treatment at any time before study inclusion; none of the patients had received antibiotics during the month preceding sample collection.

Associations between the relative abundances of bacterial taxa and fecal inflammatory biomarkers, including fecal calprotectin, EDN, and fecal IgA, were evaluated using two-sided Spearman’s rank correlation coefficients (ρ). Differential abundance between healthy controls and children with non-IgE-mediated food allergy was assessed using the two-sided Mann–Whitney U test. For differential-abundance analyses involving multiple taxa, *p*-values were adjusted using the Benjamini–Hochberg false discovery rate procedure. FDR-adjusted *q*-values < 0.05 were considered statistically significant.

All statistical analyses and visualisations were performed in R studio 2026.04 and Graphpad v 9.4. ChatGPT (OpenAI) was used to assist with language editing and formatting of statistical descriptions, while all statistical analyses and scientific decisions were conducted by the authors.

### 4.4. DNA Extraction

Fecal samples were collected from pediatric patients recruited at Marie Curie Emergency Children’s Hospital and stored at −80 °C until processing. All samples were obtained before the initiation of the food elimination diet. Microbial genomic DNA was extracted from approximately 200 mg of stool using the QIAamp DNA Stool Mini Kit (QIAGEN, Hilden, Germany) according to the manufacturer’s protocol, including inhibitor removal steps optimized for complex fecal matrices. DNA quantity and purity were assessed using spectrophotometric and fluorometric methods prior to amplification.

### 4.5. Gut Microbiome Sequencing and Bioinformatic Analysis

Bacterial community profiling was performed by amplification of the V3–V4 hypervariable regions of the 16S rRNA gene using universal primers compatible with Illumina overhang adapters (341F/806R). PCR amplification was conducted under optimized cycling conditions, and amplicons were purified using magnetic bead-based cleanup. Indexed sequencing adapters were added in a second PCR step, followed by purification and quantification. Libraries were pooled at equimolar concentrations and sequenced on an Illumina MiSeq platform (Illumina, San Diego, CA, USA) using paired-end 2 × 300 bp chemistry. Negative extraction controls and no-template PCR controls were included to monitor contamination. Sequencing quality was assessed using standard Illumina metrics.

Raw paired-end FASTQ files were processed using the DADA2 package implemented in R (4.6.0. version for Windows). Forward and reverse reads were imported and subjected to quality assessment using the plotQualityProfile() function. Low-quality regions and sequencing artifacts were removed using the filterAndTrim() function with the following parameters: truncLen = c(280,220), maxN = 0, maxEE = c(2,2), truncQ = 2, and removal of PhiX contamination enabled (rm.phix = TRUE). Reads passing quality filtering were retained for downstream analyses.

Sequencing error rates were estimated independently for forward and reverse reads using the learnErrors() function. Amplicon Sequence Variants (ASVs) were inferred using the DADA2 denoising algorithm through the dada() function, allowing exact sequence resolution without clustering into operational taxonomic units (OTUs). Paired-end reads were subsequently merged using the mergePairs() function based on sequence overlap.

An ASV table was generated using makeSequenceTable(), followed by chimera removal using the removeBimeraDenovo() function with the consensus method. The resulting non-chimeric ASV table represented the final high-confidence microbial feature matrix used for taxonomic assignment and downstream analyses.

Taxonomic classification was performed using the SILVA v138.1 reference database through the assignTaxonomy() function. Species-level annotation was additionally performed using the SILVA species assignment database with the addSpecies() function where possible. Taxonomic assignments included hierarchical classification at phylum, class, order, family, genus, and species levels.

Relative abundance analyses were conducted by aggregating ASVs according to taxonomic rank. Phylum-, genus-, and species-level abundance tables were generated and normalized to relative abundance percentages. Visualization of microbial composition was performed using the ggplot2 and pheatmap packages. Heatmaps representing relative abundance profiles across samples were generated using log10-transformed abundance matrices and hierarchical clustering.

For multi-sample analyses, ASV abundance matrices and taxonomy tables were integrated with sample metadata to enable comparative analyses between experimental groups. Diversity analyses, including alpha and beta diversity metrics, were performed on normalized ASV tables. Principal Coordinate Analysis (PCoA) and clustering approaches were used to evaluate microbial community structure and identify microbiome signatures associated with non-IgE-mediated food allergy. The generated ASV tables, taxonomy assignments, and abundance matrices were exported in CSV format for downstream statistical analysis and figure generation.

Sequencing quality was evaluated before downstream analysis. Across samples, sequencing depth ranged from approximately 14,500 to over 33,000 paired-end reads per sample, with average Phred quality scores above Q35, indicating excellent sequencing quality. Paired-end reads (2 × 300 bp) were quality filtered, trimmed to remove low-quality regions and sequencing adapters, and merged prior to downstream analyses. Chimeric sequences were identified and removed during the DADA2 denoising workflow. Amplicon sequence variants (ASVs) detected in extremely low abundance or lacking bacterial taxonomic assignment were excluded from subsequent analyses. All samples passed the predefined quality-control criteria and were retained for downstream statistical analyses.

## 5. Conclusions

The present exploratory study, including pediatric patients with non-IgE-mediated food allergy, suggests that intestinal inflammation in pediatric non-IgE-mediated food allergy is associated with specific alterations in gut microbial composition rather than a generalized loss of diversity. Several differences between patients and healthy controls were observed in the composition of the intestinal microbiome. Moreover, a positive family history of atopy was significantly more frequently reported in patients with non-IgE-mediated food allergy, compared with controls.

Higher mean relative abundances of *Bacteroides*, *Faecalibacterium*, *Alistipes*, *Parabacteroides*, *Escherichia*, and *Sutterella* were identified in patients, compared with healthy controls, while several other genera (*Escherichia–Shigella*, *Agathobacter*, and *Enterococcus/Streptococcus)* were detected exclusively in the group of patients. Conversely, healthy controls showed higher mean relative abundances *of Pseudobutyrivibrio*, *Roseburia*, *Bifidobacterium*, *Collinsella*, *Clostridium*, *Eubacterium*, *Streptococcus*, and *Barnesiella*. Despite the differences observed in the composition of the intestinal microbiome between patients and controls, the level of statistical significance was not maintained after the correction for multiple testing was applied.

Elevated fecal levels of calprotectin, zonulin, lactoferrin, IgA, α1-antitrypsin, and EDN indicated widespread mucosal immune activation. No single bacterial phylum presented a consistent association with increased fecal calprotectin levels. Shannon diversity was significantly higher in patients compared to controls and also in the subgroups of patients with elevated levels of fecal calprotectin, a positive history of exposure to antibiotics, or concomitant atopic dermatitis. Moreover, in patients with increased levels of fecal calprotectin and EDN, an increased abundance of *Veillonellaceae* and decreased abundances of *Bifidobacteriaceae* and *Lachnospiraceae* were observed. The genus-level analyses further revealed that *Veillonella* and *Escherichia-Shigella* abundances were significantly associated with higher levels of both fecal calprotectin and EDN, whereas beneficial commensal taxa, including *Faecalibacterium*, exhibited inverse associations with these inflammatory markers. The identification of inflammation-associated microbial endotypes highlights potential microbiome-derived biomarkers and provides new insights into disease heterogeneity and host–microbiome interactions in non-IgE-mediated food allergy.

## Figures and Tables

**Table 1:** Demographic, perinatal, familial, and environmental characteristics of healthy controls and children with non-IgE-mediated food allergy. Data are presented as *n* (%), mean ± standard deviation (SD), or median (Q1–Q3), as appropriate. Categorical variables were compared using Fisher’s exact test, and maternal age was compared using Welch’s independent-samples *t*-test. Percentages were calculated based on the available observations for each variable; therefore, denominators may differ because of missing data. No statistical comparison was performed for variables with insufficient distributional information or no variability in either group. A *p*-value < 0.05 was considered statistically significant and is shown in bold. IQR, interquartile range; NR, not reported; NC, not calculated.

Variable	Controls (N = 15)	Patients (N = 30)	*p*-Value
Background			**0.038**
—Urban	15 (100%)	22 (73.3%)	
—Rural	0 (0%)	8 (26.7%)	
Maternal age, mean ± SD	30.45 ± 3.22	31.20 ± 4.52	0.526
Child age, median (Q1–Q3)	5.0 (3.0–6.0)	2.5 (1.0–6.75)	NC
Sex			0.530
—Female	6 (40.0%)	16 (53.3%)	
—Male	9 (60.0%)	14 (46.7%)	
Antibiotic exposure during pregnancy	2 (13.3%)	9 (30.0%)	0.288
Smoking exposure during pregnancy	4 (26.7%)	4 (13.3%)	0.410
Premature birth	0 (0%)	0 (0%)	NC
Cesarean delivery	10 (66.7%)	19 (63.3%)	1.000
Antibiotic treatment in maternity	0 (0%)	5 (16.7%)	0.153
Formula feeding in maternity	10 (66.7%)	21 (70.0%)	1.000
Ever breastfed	12 (80.0%)	26 (86.7%)	0.670
Exclusively breastfed	5 (33.3%)	11 (36.7%)	1.000
Breastfeeding duration, median (IQR)	NR	4.5 (12)	NC
Positive family history of atopy	2 (13.3%)	15 (50.0%)	**0.023**
Presence of siblings	9 (60.0%)	15 (50.0%)	0.752
Higher maternal education	15/15 (100%)	24/28 (85.7%)	0.280
Higher paternal education	15/15 (100%)	21/29 (72.4%)	**0.037**
Low income	0/15 (0%)	7/28 (25.0%)	0.077
Pets at home	7 (46.7%)	8 (26.7%)	0.200

**Table 2:** Clinical Manifestations and Biologic Parameters of Children with Non-IgE-Mediated Food Allergy.

Variable	N (%)
Vomiting	8 (26.6%)
Regurgitation	9 (30.0%)
Diarrhea	12 (40.0%)
Haematochezia	6 (20.0%)
Constipation	2 (6.6%)
Food refusal	7 (23.3%)
Failure to thrive	11 (36.6%)
Atopic dermatitis	12 (40.0%)
Anemia	3 (10.0%)
Vitamin D deficiency	4 (13.7%)
Vitamin D insufficiency	9 (31.0%)

**Table 3:** Levels of fecal markers in patients with non-IgE-mediated food allergy.

Biomarker	Mean ± SEM	Median (IQR)	% Elevated
EDN (ng/g)	1200.82 ± 257.31	766.86 (1622.95)	33.30%
Fecal IgA (µg/g)	2318.83 ± 310.9	1990 (2082.5)	54.50%
Fecal calprotectin (µg/g)	309.05 ± 96.03	76.3 (197.12)	44.80%
Fecal zonulin (ng/mL)	78.91 ± 14.58	57.88 (53.32)	10.00%
Fecal lactoferrin (µg/g)	47.08 ± 10.98	13.7 (58.8)	65.50%
Fecal α-1 antitrypsin (µg/g)	587.61 ± 98.98	425.3 (351.7)	58.60%

**Table 4:** Comparison of the relative abundance of the 20 most abundant bacterial genera between healthy controls and children with non-IgE-mediated food allergy. Relative abundances are presented as mean ± standard deviation (SD) and were calculated as the percentage of reads assigned to each genus relative to the total number of reads per sample. Differential abundance between healthy controls (*n* = 15) and children with non-IgE-mediated food allergy (*n* = 30) was assessed using the Mann–Whitney U test. Fold changes are expressed as log_2_ fold change (log_2_FC) calculated as Allergy/Healthy. Multiple testing correction was performed using the Benjamini–Hochberg false discovery rate (FDR) method. Positive log_2_FC values indicate genera enriched in the allergy group, whereas negative values indicate genera enriched in healthy controls. NS, not statistically significant after FDR correction.

Genus	Healthy (%), Mean ± SD	Allergy (%), Mean ± SD	log2FC (Allergy/Healthy)	Mann–Whitney *p*	FDR (*q*)
*Bacteroides*	11.19 ± 13.55	30.24 ± 27.57	1.43	>0.05	NS
*Faecalibacterium*	5.83 ± 8.07	20.15 ± 20.59	1.79	>0.05	NS
*Pseudobutyrivibrio*	18.51 ± 25.84	0.29 ± 1.39	−6.00	>0.05	NS
*Roseburia*	11.77 ± 18.62	3.20 ± 10.58	−1.88	>0.05	NS
*Bifidobacterium*	11.68 ± 19.11	2.74 ± 6.90	−2.09	>0.05	NS
*Collinsella*	8.88 ± 22.58	0.95 ± 2.82	−3.22	>0.05	NS
*Clostridium*	8.56 ± 21.92	2.12 ± 8.08	−2.01	>0.05	NS
*Escherichia–Shigella*	0.00 ± 0.00	8.19 ± 16.90	↑	>0.05	NS
*Alistipes*	2.07 ± 4.69	4.88 ± 7.87	1.24	>0.05	NS
*Eubacterium*	2.98 ± 8.42	0.31 ± 1.30	−3.25	>0.05	NS
*Parabacteroides*	1.35 ± 2.58	2.97 ± 5.63	1.14	>0.05	NS
*Streptococcus*	2.62 ± 7.17	0.41 ± 1.37	−2.68	>0.05	NS
*Prevotella*	1.17 ± 3.47	1.92 ± 4.50	0.71	>0.05	NS
*Cronobacter/Enterobacter/Escherichia/Salmonella group*	0.00 ± 0.00	1.66 ± 4.94	↑	>0.05	NS
*Collinsella/Coriobacterium*	0.00 ± 0.00	1.66 ± 5.12	↑	>0.05	NS
*Escherichia*	0.34 ± 1.03	1.48 ± 5.12	2.12	>0.05	NS
*Sutterella*	0.72 ± 1.42	1.43 ± 3.22	0.99	>0.05	NS
*Agathobacter*	0.00 ± 0.00	1.17 ± 3.91	↑	>0.05	NS
*Barnesiella*	0.81 ± 2.37	0.19 ± 0.70	−2.09	>0.05	NS
*Enterococcus/Streptococcus*	0.00 ± 0.00	0.78 ± 3.10	↑	>0.05	NS

↑ indicates genera detected in the allergy group but absent from the healthy control group; therefore, the log_2_ fold change could not be calculated because the mean relative abundance in the denominator was zero.

## Data Availability

The 16S rRNA amplicon sequencing data generated in this study have been deposited in the NCBI Sequence Read Archive (SRA) under submission SUB16061512 (BioProject PRJNA1447955), Mendeley Data repository https://doi.org/10.17632/9skgh53n6z.1. The accession numbers will be made publicly available upon completion of the SRA processing and prior to final publication. Due to ethical and legal restrictions related to sensitive clinical information from pediatric participants, individual-level clinical datasets are not publicly available. De-identified data supporting the findings of this study may be made available from the corresponding author upon reasonable request and subject to approval by the relevant institutional ethics committee.
